# Predictors of higher education dropout intention in the post-pandemic era: The mediating role of academic exhaustion

**DOI:** 10.1371/journal.pone.0327643

**Published:** 2025-07-08

**Authors:** Bárbara Gonzalez, Teresa P. Mendes, Ricardo Pinto, Sónia V. Correia, Sara Albuquerque, Paula Paulino

**Affiliations:** 1 Lusófona University, HEI-Lab: Digital Human-Environment Interaction Labs, Campo Grande, Lisboa, Portugal; 2 CICPSI, Faculty of Psychology of the University of Lisbon, Lisboa, Portugal; 3 CIDEFES- Centro de Investigação em Desporto, Educação Física, Exercício e Saúde, Lusófona University, Campo Grande, Lisboa, Portugal; 4 CINEICC, Faculty of Psychology and Educational Sciences of the University of Coimbra (Portugal), Coimbra, Portugal; University of Cambridge, UNITED KINGDOM OF GREAT BRITAIN AND NORTHERN IRELAND

## Abstract

**Introduction:**

The phenomenon of dropout in higher education needs the acknowledging of its multi-domain complexity. In the post-pandemic era, exhaustion may be a relevant feature affecting students. This cross-sectional study aimed primarily to test a predictive model of five domains of variables (background, academic, social, psychological, and economic) on dropout intention, in a relation mediated by academic exhaustion. Secondarily, it aimed to assess the structural invariance of this model across working status (working vs. non-working students) and residence status (living away from family’s residence vs. living in family residence). If these groups are differently affected by dropout determinants, specific dropout prevention measures should be implemented.

**Method:**

A stratified sample of 1402 Portuguese university students aged between 19 and 45 years (*M* = 22.87, *SD* = 3.64), selected through a convenience quota method, was assessed for background, academic, social, psychological, and economic variables using self-report instruments. Structural equation modelling was used.

**Results:**

The predictive model explained 51% of the variance in dropout intention. Academic exhaustion was the stronger predictor (β = 0.523, p < .001), followed by social connecteness to the campus (β = −31, p < .001), vocational difficulties (β = 0.274, p < .001), and course value (β = −0.256, p < .001). Except for the course value, and family educational level, all significant predictors had their effect on dropout intention through academic exhaustion. The model was invariant across working and residence status.

**Discussion:**

This study shows the relevance of students’ academic exhaustion experiences as a pathway through which different types of factors exert their influence on students´ dropout intentions. The invariance of the predictive model of dropout intention across different groups points the robustness of the model and the relevance of the integrated variables. The results emphasize the importance of student´s individual factors (e.g., academic exhaustion, lack of fit with the course) in dropout decisions, also stressing the role of academic institutions and of the education system in addressing this phenomenon, concerning academic workload, vocational orientation, social environment, and financing.

## Introduction

Dropout is a major concern in higher education (HE) across different countries, with an average dropout rate around 30% among OECD countries [1]. Dropout is a multifaceted phenomenon that can carry different meanings [2–4]. Some students who leave their degree program before completion may not be making a final decision, potentially taking a break, switching courses or institutions, and later returning to HE. However, for approximately 40% of the students, dropout means not returning to HE [5]. HE dropout involves negative consequences not only for students (e.g., sense of personal failure, delays financial autonomy), for families (e.g., financial burden), but also for institutions (e.g., in funding, performance, reputation drop), and society (e.g., waste of economic and educational resources) [2,6].

The COVID-19 pandemic has brought unprecedent challenges to HE students [7,8]. Public health measures to contain pandemic effects, like mandatory restrictions over social contacts and freedom to move, impacted the way university courses were delivered, hampering students’ ability to create connections with other students and institutional staff, satisfaction about education formats and students’ academic performance and psychological well-being [7,9–11]. Although since April 2023, the COVID-19 infection was no longer classified as a public health emergency [12], it is expected that changes brought by the pandemic have had a lasting and cumulative impact on the HE academic dynamics and on students’ education trajectories. Recent longitudinal studies show that students’ likelihood of dropping out increased in 2021–2023 period, compared to 2020, along with academic exhaustion levels [13]. On the other hand, study satisfaction levels that initially rose during the 2021, declined subsequently in 2022 [13,14]. In this context, gaining a better understanding of the factors that contribute to dropout rates in HE in this post-pandemic era is crucial.

### Conceptualizing students’ dropout determinants: a brief review of theoretical models

Dropout is a complex phenomenon. Over the years, researchers from different disciplines (i.e., sociology, psychology, economy) developed theoretical models that led to the identification of several relevant factors for students withdrawing from HE [2,6].

Departing from sociology, the first and perhaps the still most relevant theoretical model to examine dropout is Tinto’s [15] “Integration Model”. Conveying a dynamic longitudinal and process perspective regarding students’ dropout, Tinto [15] holds that students’ initial commitment, goals, and intentions are influenced by pre-existing characteristics like prior academic performance and personal and family background. Upon entering university, students’ interactions with academic and social environments affect their integration into these systems. Whereas academic integration is mainly reflected in students’ performance, social integration is fostered through casual peer group connections, extracurricular pursuits, and students’ interactions with faculty and other staff. Successful integration strengthens students’ goals and commitments, promoting persistence, whereas poor integration weakens them, heightening the likelihood of withdrawal. Later, focusing on economically disadvantaged populations and non-traditional students (e.g., working students, students with caregiving responsibilities), Cabrera et al. [16] and Bean et al. [17] further elaborated on the role played by financial resources and by social support outside university in students dropout decisions.

Contrarily to sociologically oriented models of HE students` dropout that highlight the concepts of academic and social integration at university, psychological models concentrate on the role of students’ attributes and behavior when making the decision to leave the HE system [18,19]. According to these authors, students enter university with pre-entry psychological traits like self-efficacy and attributions. These traits influence their interactions with the institutional environment, which trigger psychological processes such as self-efficacy assessment and coping. Successful processes enhance self-efficacy, reduce stress, and foster internal attribution and motivation, aiding academic and social integration, which positively influence students persistence and determination [19]. For psychological theories, the focus is placed on individual self-regulatory long-term processes, meaning that students dropout is driven and regulated from within [20], placing less emphasis on institutional factors. In this line, and emphasizing the longitudinal nature of dropout, Bäulke et al. [21] put forward a conceptualization of the dropout process as comprising the following phases: perception of a poor fit between students and institution, ruminations over dropping out or changing courses, and searching for information about alternatives, ultimately leading to the final decision to dropout.

On the other hand, economically motivated models focus on concepts of rational decision-making and opportunity costs versus anticipated labor market benefits, which students consider as they move along their HE trajectory [14,22]. Students will leave the educational system if anticipated advantages do not surpass perceived costs.

More recently, integrative models have emerged, namely the one developed by Behr et al [2], grounded in an exhaustive literature review regarding the levels and domains of factors that can influence students’ dropout decisions. The author classifies dropout determinants according to their level of influence and potential for change, from national level determinants (associated with the national education system like country’s education system organization or financing policy), to institutional (related to the HE institution like type of institution, and high achievement requirements), and individual students’ factors. For Behr et al. [2] students’ individual factors can be subdivided into: a) pre-study determinants like parental educational background or prior education factors (e.g., grade point average at high school) and b) study related factors, like students’ psychological characteristics, degree program satisfaction and person-environment fit. National education system factors do not seem to be ‘stand-alone’ predictors, but rather interact with other national factors and those from more institutional and individual levels.

Based on the presented theoretical models of dropout, it stands out that dropping out decisions rarely depend on only one isolated factor but are rather the result of long-term decision-making process where multiple factors may play a role. Moreover, these clusters of causes for dropping out seem to be mainly a combination of factors from different areas (e.g., personal and institutional factors), rather than covering factors from only one area.

### The Portuguese HE system

The HE system in Portugal consists of two main types of institutions: universities and polytechnics. Universities focus on research-oriented education and theoretical knowledge, while polytechnics emphasize applied sciences and professional training. The Bologna Process has significantly influenced the system, aligning degree structures with European standards through a three-cycle model: bachelors (1st cycle), masters (2nd cycle), and doctoral programs (3rd cycle). Both public and private institutions coexist within this framework, regulated by the Ministry of Education, Science and Innovation. HE can be taught in public institutions (state and foundations) and private institutions (private entities and cooperatives). The process of opening places in HE is regulated by the *numerus clausus* system, which limits the number of available places [23]. The Portuguese admission process for public institutions is conducted at the national level and relies on an application score, which is calculated using a weighted combination of upper high school grades and national examination results in core scientific subjects. Applicants can submit up to six choices of institution and study program, ranked by preference. Public HE imposes very high admission grades [24]. According to the Directorate-General for Education and Science Statistics [25], 80.6% of students were enrolled in public HE institutions. Both Public and Private HE institutions charge tuition fees. Usually, public universities have significantly lower tuition fees compared to private institutions. At public HE institutions, the annual tuition fee, both for technical higher vocational courses and for 1st and 2nd cycle courses that are legally required for professional practice, is set by each HE institution and ranges from EUR 495 to EUR 697 (2022/23) [26]. Portugal is considered a low tuition fees country [27], but it is relevant to mention the country minimum salary of 760 euros at that time. Institutions teaching other cycles are free to set their own tuition fees. Portugal is the second European Union country in which the financing of HE by the students´ family is higher, being that 68% of students count on their families as the exclusive source for financing [28]. In 2022/23, 20% of students enrolled in bachelor programs and 15% of those enrolled in masters received social scholarships. Students’ scholarships may be awarded based on social considerations, for students from underprivileged backgrounds, or on the basis of exceptional academic merit, regardless of students’ income. Students who are studying away from their usual place of residence may be eligible for housing benefits, depending on family income. Although Portugal’s expenditure per student at most educational levels and its Gross Domestic Product (GDP) per capita are both below the OECD average, it spends a larger share of its GDP per capita on education than the OECD average [29].

Regarding working status, in 2021/2022 about 31% students enrolled in Portuguese institutions worked while studying [28]. The number of students enrolled in HE has gradually increased every year, with a 24% increase since 2015/16. Concerning the pathways in Portuguese HE, is shown by an official report that only half of the of students enrolled in three-year bachelor’s programs were able to finish their degrees within four years, while approximately 29% of students left their higher education studies at some point during this period [30]. The tertiary education attainment rate among individuals aged 25–34 in Portugal was 41.5% in 2023, slightly below the EU average of 43.1%. [31]. In Portugal, since 2022, the rate of overqualification in young workers (25–34 years old) had a 2.5 percentage points increase, reaching 22.4% and reversing the slight decrease tendency that took place between 2018 and 2021 [31]. Furthermore, in 2023 there was an increase of the unemployment rate in population with higher education [32]. In Portugal, 25–34 year-old who completed a vocational upper secondary or post secondary non-tertiary program have lower inactivity rates than those with a bachelor’s or equivalent degree [29]. Given these data, research on HE and features that may influence students to persist or not in their academic path is needed in Portugal, for informed implementation of dropout preventive measures.

### Empirical review of dropout predictors: grounding the present study

Despite abundant literature on dropout, there is still no consistent agreement across different studies about the stronger role of different domains of variables – pre-study determinants, psychological, academic, social, economic, institutional – in predicting dropout [2,33]. To inform effective preventive measures of dropout within HE institutions, a clearer picture of the relative contribution of different domains of factors is essential. While Behr et al. [2], following George et al. [34] considers that the decision to leave the university without obtaining a degree is driven mainly by students’ personality and academic self-concept (individual level) and less by external factors (institutional side, national system level), research that covers the different domains of predictors is scarce. To identify which students’ face particular challenges and may be at risk of dropping out, analysing students’ intention to dropout can be particularly useful [35,36]. Dropout intention is considered a reliable proxy for dropout [37,38]. A recent systematic review has suggested that future research should investigate the intention to dropout, understanding it as a complex process, privileging different levels of characteristics in the analysis [36]. Moreover, research on dropout determinants must take in consideration the new educational realities caused by the COVID-19 pandemic. Pre-existing financial and education disparities, as well as inequality of opportunities for success in HE, may have been heightened [14,28].

Based on the presented theoretical models of dropout we organized predictors of dropout intention found in literature in five different domains: background, academic integration, social integration, psychological, and economic; and for each domain we selected specific variables. Examining the joint contribution of different domains of predictors in a single complex predictive model can help us understand which of these groups of variables has greater influence in dropout processes in this post-pandemic era, as well as unravel possible pathways through which these links with dropout intention may occur.

### Background variables

Family educational level. In general, students from non-academic family backgrounds (e.g., family members that did not pursue tertiary education – i.e., education after high school completion) in many European countries show higher dropout rates [39]. The higher the level of parental education, the better the students’ performance at university and the lower the likelihood of dropping out [16,40]. Students from non-academic families are expected to be more heavily discouraged by experiencing academic difficulties, since they do not possess compensatory resources and support from academically oriented families and significant others outside of the HE institution [39]. However, there are studies in which family educational level does not significantly relate to dropout [41–43].

Pre-entry grade. The students grade point average at high school is regarded as an important indicator of students ability to meet the performance level required by the HE system, constituting one of the most consistent factors affecting dropout found in literature [2,42]. Students with higher entry grades have previously acquired knowledge and academic competences that constitute a protective factor against dropout [41,44]. Students who enter university with lower grades tend to perform poorly in their university exams, placing them at greater risk of dropout [5,45].

Course as first option. In Portugal’s HE admissions process, which is based on a *numerus clausus* system, students with higher grades are more likely to enroll in their preferred courses. Those who apply to public institutions, if they do not gain admission to their first choice, may be able to do so in one of the other five choices listed. This *numerus clausus* system prevents other students from getting on their preferred study program. Not being able to enter their course of choice, might lead to a sense of low self-efficacy in comparison with their colleagues, which has effects on well-being and academic performance [46,47]. While not attending first-choice course was found as a significant predictor of dropout [6,41], some inconsistencies are found in literature [48].

### Academic integration variables

Satisfaction with education. Dissatisfaction with the quality of the students’ experience is outlined as one of the main reasons to dropout [5,10,49]. Students dissatisfied with class organisation, schedules and teacher availability are more likely to leave [13,46]. COVID-19 pandemic had a profound impact on teaching and learning processes and outcomes [50], with students reporting increased levels of complaints and dissatisfaction with HE experiences [10].

Academic self-efficacy. Students with higher perceived academic abilities and academic performance expectancy have a lower intention to dropout [51,52], and self-efficacy expectations were one of the most relevant predictor variables to explain the intention to remain in HE [53,54]. In the German context, difficulties with the study content were considered the most important reason for dropout [6], however, this result is inconsistent with other studies results [34,43].

Grade performance. Performance deficits are interpreted as a mismatch between students’ abilities and the requirements of their study program. Across many HE systems, students’ performance is one of the most cited reasons for students dropout [54,55]. Previous research has found that academic difficulties and a lack of academic control increase the likelihood of dropping out [56], and academic achievement in the first year was considered the most relevant predictor of dropout [33,46].

Course value. In the competitive global knowledge economy and with governments concerned about ensuring a highly skilled workforce, HE has been proposed as a solution to meet labour force needs [57]. Course value implies an orientation towards future, the probability of a successful integration in the work market. It may be identified with the utility value, one of the elements that integrate the subjective task value, within the Situated Expectancy Value Theory [58], conceptualized in terms of how well a particular task fits into an individual’s present or future plans. Research with higher education students suggested that students’ use of effective cognitive learning strategies is driven by the perceived utility value in relation to students’ learning goals [59] and studies have shown the negative relation of utility value with dropout intention [60,61]. Course value was considered one of the most relevant predictors of dropout intention, and students with lower satisfaction with the course were also at higher risk of presenting academic burnout [53] and higher dropout intentions [5,44,54]. Each year, students face a critical decision regarding their HE, as they weigh opportunity costs against anticipated labour market benefits. This perception is significantly affected by external factors such as financial crises, business cycles and pandemics [14], so the role of this variable needs to be further explored in this post-pandemic context.

Vocational difficulties. Students’ commitment to the academic content of the study program can also be defined as students’ intellectual development, which is described by Tinto [40] as part of academic integration. Students’ subjective level of uncertainty about their belonging within their domain of study significantly predicted students’ dropout intentions above and beyond academic performance [34,62]. This feature has a direct effect on academic integration, fundamentally involving a reduction in class attendance and social activities and is named as a factor contributing to academic success or failure [5]. A lack of interest in the field of study is cited as one of the most prevalent reasons for why students chose to abandon their studies [6], and satisfaction with the area of study was full mediator of the relationship between outcome expectations and dropout intention [52].

### Social variables

Social connectedness to the campus. Students’ interaction with fellow students is one sub-dimension of social integration as defined by Tinto [15], and a high degree of social integration is conditional upon the quality and the quantity of these relationships. Fellow students provide opportunities for informal academic collaboration and sources of emotional and social support. Friendships guide students in navigating the transition into their institution and enhance students’ sense of belonging [43,63]. Social connectedness enhances social life, promoting friendships, and minimizing homesickness [64], and social integration, apart from its importance for students’ well-being, can also act as a buffer when problems with academic performance occur [65]. Some studies suggest that HE social satisfaction had a higher influence on dropout intention than the perception of academic performance [66], but this result needs further support.

Difficulties in adaptation to institution. Students’ interaction with faculty is one sub-dimension of social integration as defined by Tinto [15]. Faculty members and other members of the HE institution provide students with educational and learning contexts that are supportive and create meaningful academic experiences, resulting in higher attachment and reduced dropout [40,67]. Previous studies have found a positive association between (formal and informal) teacher- relationships and students’ academic motivation, achievement and persistence [68].

### Psychological variables

Well-being. Well-being plays an important role in students’ academic performance and drop-out rates. Studies report that a substantial number of students in HE is dealing with well-being issues such as psychological and emotional distress, feelings of anxiety and depression, and an increased risk of burnout [8,28]. Mental health issues represent a significant burden for students worldwide and high-performance pressure and performance drive are often used to explain lower levels of well-being of HE students [69–71]. This situation became more pervasive with pandemics, with remote teaching and contact restrictions [8,72,73]. It is also known that HE students often do not seek help form formal sources of support within or outside the educational context [72,73]. High subjective well-being can be seen as a protective factor against dropout as it relates to important study-relevant factors such as academic procrastination and performance [74].

Autonomy difficulties. The HE experience represents for many a significant leap to more independence and personal and academic autonomy, related to the complexities of transitioning from adolescence to early adulthood [75,76]. Transferring from the controlled environment of school and family home to undertaking personal responsibility for academic, financial and social aspects of life is challenging [45,76]. Some students may be/feel unprepared to deal with the required tasks related to autonomous living and autonomous learning (e.g., taking on responsibilities on their own daily lives, managing time, stress and studies), and may need to develop new coping mechanisms to manage the demands and/or perceived lack of support from their institution [77,78]. Past studies show that autonomous motivation positively predicts academic achievement and negatively predicts dropout intentions [78].

Satisfaction with social support. Several studies point the positive effect of this variable on low dropout intentions [44,66]. A key factor for optimum integration, especially in the first year of university, is the support students receive from their families [79]. Social support is one important predictor of perceived stress among HE students, because in addition to the emotional support and instrumental assistance, it reaffirms the relevance of the student’s membership in the academic environment, building on core motivational values [65]. Social support satisfaction and adaptive coping are associated with increased levels of academic engagement [80].

### Economic variables

Economic difficulties. Students from lower socioeconomic backgrounds are more vulnerable to dropout [14,81]. Although gaps in access to HE between high- and low-income students have decreased, gaps in completion between these groups of students tend to persist [28,40]. Students without a tertiary background tend to depend on public funds or on their job earnings rather than on their family support. On cross-country average, 42% of students receive national public support (grants, loans, or scholarships). This type of support, on average, is about 42% of the recipients’ total monthly income [35]. Financial aid is important not only because it equalizes opportunities between high- and low-income students, but also because it facilitates the integration of students into the academic and social components of the institution and influences their commitment to stay in HE [43,82].

Decrease in financial conditions due to the pandemic. On average, 23% of students report a (very) negative impact on their study financing due to the pandemic [28]. This percentage varies by country, it’s 36% in Portugal, the highest among Central European countries. Rising expenses due to inflation in the years 2022/23, online learning equipment costs, and increased health costs further increased financial strain [28]. Moreover, students from financially disadvantaged families were particularly affected by such negative impacts of the pandemic, through the loss of students’ jobs, reduction of family support, or difficulties obtaining public support, and were found to be at a higher risk of dropping out in post-pandemic [14,28].

### Predicting students’ dropout intention: the missing link of academic exhaustion

HE students face multiple stressors related to incessant academic demands, and the continuous management of diverse responsibilities [83,84]. Over the last two decades, researchers have increasingly drawn attention to the alarming prevalence rates of burnout among HE students [85], as well as to the association between burnout and intentions to dropout from HE [65,86]. Dropout is rarely the result of short-term or spontaneous decisions, but rather of a long decision-making process, where multiple conditions and problems accumulate, leading students to leave HE without a degree [3,87].

Emotional exhaustion (i.e., individual’s feelings of being emotionally exhausted and depleted of emotional resources), cynicism (i.e., attitude of indifference towards academic activities), and reduced professional efficacy (i.e., lack of confidence in one’s academic abilities) are considered the three components of burnout and tend to grow over time [8]. Schriek et al. [13] has recently shown, in a large sample of German university students, that both emotional exhaustion and dropout intentions have increased in 2021–2023 compared to 2020. Exhaustion can seriously affect academic performance [88]. Academic exhaustion has been positively associated with dropout intentions, mostly in cross-sectional studies [80,86,89]. A recent longitudinal study has shown that emotional exhaustion at the beginning of the semester predicted students’ intentions to dropout from university at the end of the semester. Also, while cynicism develops last in the burnout process of students, ultimately triggering dropout decisions, academic exhaustion can be considered an initial indicator, operating as strong predictor for dropout intentions [8]. Therefore, promptly identifying signs of academic exhaustion could be beneficial in providing students with timely assistance.

Considering dropout as an evolving self-regulatory long-term process characterized by cognitive and behavioral patterns, in which ruminations about dropping out evolve after perceiving a poor fit with the institution [21], academic exhaustion may be the consequence of these ruminations, and a proximal determinant of dropout intention. So far, few studies have tested academic exhaustion as a mediator variable in the relationship between factors such as social support, general distress [80], and fear of COVID-19 [89] and dropout intention. In the workplace, several studies have found that exhaustion mediated the effect of relevant variables on turnover intention (i.e., conscious and deliberate desire to leave their current organization or job), namely role conflict [90], and abusive supervision [91]. Therefore, as a possible ultimate proxy of dropout intention, it makes sense to test a model in which exhaustion is a mediator between a set of different domains of variables, and dropout intention.

### Working-students and students away from family’s residence

In recent decades, there has been a growing democratization of access to HE which has been correlated with a progressive differentiation in the profiles of students entering and attending HE, in terms of trajectories, motivation, and academic competencies [28,92]. Predictors of dropout intentions may have different relevance for sub-groups of students, whose (dis)advantages may have been amplified by the pandemic [14,28]. Concerning working-students, and students living away from home to attend university, most of the studies focuses on comparing dropout rate of these populations with the one of ‘traditional’ students [5,28,92–95], and not on the potentially different effect of dropout predictors in these students. Previous research has shown that undergraduate students living away from home may be at higher risk of dropping out of HE [45,79] but results have been inconsistent [51]. While studying outside family homes may give HE students greater opportunities to exercise autonomy and responsibility in their lives – which is aligned with the quests of emergent adulthood [75], living away from home brings significant added challenges that may interfere with their academic adaptation, ultimately leading to dropout. Pertaining to variables that may have different impact on the experience and challenges of persisting on HE, some research has shown that social integration, especially with academic staff, may be particularly relevant for working students [40,67,95], as the classroom may be the only place on campus in which they spend any appreciable time [40]. For those studying away from home, there is also a greater risk of homesickness and loneliness as this is when most students are separated for the first time from their families [79], and availability of family support may compensate for a lack of social integration at university, one of the important predictors of dropout.

### The present study

According to a Behr et al. [2], former studies mostly emphasise so-called ‘hard’ factors, which are not influenceable by institutions (e.g., age, social background, school grades). However, research would in fact benefit from focusing more on the so-called ‘softer’ or attitudinal-based factors, such as satisfaction or integration, which may all be positively affected by the institution [4], and can be regarded as modifiable. Thus, there is the need of a broader and integrated understanding of the factors that contribute to dropout rates in HE, in the post-pandemic context, including factors related to the time before and during HE studies, as well as ‘softer’ attitudinal-based and university modifiable factors, along with non-modifiable factors (e.g., family educational background).

Much of previous research tested predictive models of dropout for a whole sample [44,66]. However, acknowledging previous evidence of the influence of students’ working status, and students living situation, on students’ dropout intention [28,79,93], it is important to assess if these groups are differently affected by dropout determinants, in which case specifically designed dropout prevention programs should be implemented.

Dropout it is not exclusive of students entering HE for the first time [5,79,94]. However, most of the abovementioned studies have purely convenience samples, from one or few HE institutions, and mainly of 1st year students [51,54,66]. The present study focuses on dropout intention throughout the entire academic course. In the same vein, many of the mentioned studies are based on small data sets and restrict their analysis to specific academic fields and/or to one university [37,45]. Our sample also includes a diverse sample of students attending HE institutions across Portugal (public and private, university and polytechnics, and different regions). Highlighting the multifaceted nature of HE dropout, and the fact that various factors interact in intricate ways we opted to analyse our data based on complex analytical tools (structural equation modelling). Moreover, the decision to quit studies (leaving studies before degree completion) and changing a major (change course and/or institution while continuing the system), may fulfil different functions and may be associated with different consequences for the individual [2,21]. This study focuses on students who intend to leave HE entirely, rather than just transferring or switching courses/institutions, considering the importance of taking a broader societal perspective of this phenomenon [2], instead of investigating dropout at a micro level (which matters specially to institutions).

Our aims are: 1) to test a predictive model of dropout intention, more precisely, to test the direct and indirect links, between five different domains of variables (background, academic integration, social integration, psychological and economic) and dropout intention, through academic exhaustion, in a sample of Portuguese HE students. 2) assess the structural invariance of this model across working status (working vs. non-working students) and residence status (living away from family’s residence vs. living in family residence).

Based on previous literature, we expect that these variables will significantly predict dropout intention (H1), with academic exhaustion acting as a mediator (H2), and academic exhaustion having the greater effect on dropout intention (H3). Pertaining to our secondary goal, it is more exploratory, as the lack of consistency across reviewed studies does not allow us to specify hypotheses.

## Method

### Participants

From an initial matrix of Region NUTs II, the respondents were selected through a convenience quota method, based on a matrix that crosses Sex and Age variables (based on 2021 Census). The type of institution (university/polytechnic) and the education system (public/private) were monitored throughout the data collection, based on data from the Directorate-General for Statistics of Education and Science (DGEEC). Those eligible to participate in the study were: students currently attending a Portuguese HE institution (public or private) from first to last year of studies required for certified practice (up to 7 years), currently living in Portugal, and Portuguese speaking. Being an Erasmus student was an exclusion criterion.

The study sample was composed of 1402 university students (ages ranging from 18 to 45 years; *M* = 22.83; *SD* = 2.73) mostly from Lisbon (36.2%) or the north region of Portugal (33.8%). The majority of the participants were female (54.4%), single (83.5%), pursuing a bachelor’s degree (65.3%) in a public (79.8%) university (76.4%). The students were enrolled in courses from various subject areas: science and technology (49.1%) social and economic sciences (29.9%), languages and humanities (12,3%) and arts (8.7%). The students were mostly on their first (23.7%); second (29.8%), or third (26.3%) academic year. In 81.2% of the cases, students attended their first option course. The majority of students have parents without a college degree (60.8%). Half of students that comprised our sample (50.6%) was living away from their family’s residence. Families were the primary source of income for students (65.5%), and about 40% of the students’ combined studies and work, working on average 24.9 hours per week. Moreover, 20% of students reported receiving economic support from the government. Monthly education expenses were on average 538 euros. Regarding personal or family income, 43.5% stated it had become slightly worse or considerably worse after the pandemic.

A priori power analysis was conducted using G*Power 3.1 to determine the minimum required sample size for the study. Assuming a small effect size (f² = 0.02), an alpha level of.05, a power of 0.90, and 20 predictors (including control variables), the analysis indicated that at least 1323 participants would be necessary. The final sample of 1402 participants exceeds this threshold, ensuring adequate statistical power to detect small effects.

### Instruments

**Dropout intention**

This variable was assessed with three of the four items of the subscale of dropout intention (e.g., “I am thinking of leaving higher education.”) of the Screening Instrument for Students At-Risk of Dropping out from HE [46]. Several researchers recommended differentiating between quitting studies completely and changing a major [2,3,21]. In the present paper, we examine the process of dropout in terms leaving the HE system. Each item was answered on a 5-point Likert scale, ranging from 1 (never) to 5 (always). Mean ratings were calculated, with higher scores indicating higher risk of dropping out from HE. In the current sample, the Cronbach’s alpha was .86.

**Academic Exhaustion**

This variable was assessed with the four-item Academic Exhaustion subscale (e.g., “I feel exhausted due to my course activities.”) of the Screening Instrument for Students At-Risk of Dropping out from HE [46]. Each item was answered on a 5-point Likert scale, ranging from 1 (never) to 5 (always). Mean ratings were calculated, with higher scores indicating higher levels of academic exhaustion. In the current sample, the Cronbach’s alpha was .83.

### Sociodemographic and background variables

The following variables were assessed: gender, age, marital status, national region, education system (university/polytechnic), type of institution (public/private), area of knowledge (Science and Technology, Social and Economic Sciences, Languages and Humanities, Arts), study cycle (bachelor, master), academic year, pre-entry grade (mean score of high school conclusion), family educational level (students were asked about the maximum education level attained by mother or father), whether the degree they were in was their first choice, working status (if they were working while studying; and if so, the mean number of working hours per week), and residence status (if they are living and studying away from home).

### Academic integration variables

Satisfaction with education was assessed with the four items subscale (e.g., “I am satisfied with the education I am receiving at this university.”) of the Screening Instrument for Students At-Risk of Dropping out from HE [46]. Each item was answered on a 5-point Likert scale, ranging from 1 (never) to 5 (always). Mean ratings were calculated, with higher scores indicating higher levels of satisfaction with education. In the current sample, the Cronbach’s alpha was.85.

Academic self-efficacy was assessed with the four items of the Academic Self-Efficacy Scale [96] (e.g., “I feel that I am able to focus on the subjects I study”). Each item is answered on a 5-point Likert-type scale ranging from 1 (strongly disagree) to 5 (strongly agree). Mean ratings were calculated, with higher scores indicating better academic self-efficacy. In the current sample the Cronbach’s alpha was.88.

Grade performance was assessed with the Self-perception of Academic Performance Scale [97], a single-item scale that asks students to posit their grades in comparison with the minimal grade required to pass, in a 5-point Likert-type scale ranging from 1 (much higher than the minimal grade) to 5 (much lower than the minimal grade). Students were given the option to skip this question due to the lack of assessment elements following their recent entry into HE. In such cases, the response was excluded from further analyses, which applied to 25 students.

Course value, i.e., the degree to which the perceives the course he/she is attending as useful and valuable for personal and future goals, was assessed with the Perception of Course Value in Face of Personal and Future Goals Scale [96]. The scale has three items (e.g., “What I learn at the University is important to perform my professional activity throughout my life”), answered on a 5-point Likert-type scale from 1 (strongly disagree) to 5 (strongly agree). Mean ratings were calculated, with higher scores indicating higher course value. In the current sample, the Cronbach’s alpha was.85

Vocational difficulties were assessed with one item of the Instrument for Exploring Difficulties in Academic Adaptation [98]. Students were asked to assess to what extent they have been experiencing vocational difficulties (e.g., not liking the degree, finding out that the degree is not what was expected, job prospects), in their current life as HE students. The students’ answers were graded from 1 (no difficulties) to 5 (several difficulties).

### Social integration variables

Social connectedness to the campus was assessed with the Social Connectedness Scale (SCS) [99]. This unidimensional 8-item scale (e.g., “I feel very distant from other students”) assesses HE students’ personal sense of belonging on campus and connectedness to the campus community. Students indicated on a 6-point Likert-type scale (1 = strongly disagree; 6 = strongly agree). All items are reversed, and higher mean ratings indicate higher social connectedness to the campus. In the current sample the Cronbach’s alpha was.92.

Difficulties in adaptation to the academic institution were assessed with one item of the Instrument for Exploring difficulties in academic adaptation [98]. Students are asked to assess to what extent they have been experiencing difficulties in the adaptation to the institution (e.g., spaces and services, relationship with teachers). The students’ answers are graded from 1 to 5, from “no difficulties” to “several difficulties”.

### Psychological variables

Satisfaction with social support was assessed with the Social Support Satisfaction Scale (ESSS) [100]. This 15-item scale (e.g., “Even in the most embarrassing situations, if I have an emergency, I have several people I can turn to”, “I am satisfied with the way I relate to my family”) aims to capture four areas of social support: friends, intimacy, family and social activities. Each item was answered on a 5-point Likert-type scale, ranging from 1 (never) to 5 (always). In the present study we used the total averaged score. The higher the mean ratings, the greater the satisfaction with social support. In the current study, the Cronbach’s alpha for the overall scale was.85.

Well-being was assessed with the 3-item Emotional Wellbeing subscale of the Mental Health Continuum Short Form (MHC-SF) [101,102]. This subscale assesses positive emotion and life satisfaction, considered hedonic well-being. Students rated the frequency of feelings such as happiness, interest in life and satisfaction (e.g., “How often did you feel happy?”), in the past month on a 5-point Likert-type scale from 1 (once or twice in the last month) to 5 (every day). Mean ratings were calculated, with higher scores indicating better levels of well-being. In the current study, the Cronbach’s alpha was.90.

Autonomy difficulties were assessed with one item of the Instrument for Exploring Difficulties in Academic Adaptation [98]. Students were asked to assess to what extent they have been experiencing autonomy difficulties (e.g., living alone, trusting oneself, managing stress, taking on responsibilities alone, missing the family) in their current life as HE students, in a 5-point Likert-type scale from 1 (no difficulties) to 5 (several difficulties).

### Economic variables

Economic difficulties were assessed with one item of the Instrument for Exploring Difficulties in Academic Adaptation [98]. Students were asked to assess to what extent they have been experiencing economic difficulties (e.g., paying for daily expenses, paying for course materials, paying the tuition fees, not having a scholarship). in their current life as HE students. The students’ answers are graded from 1 to 5, from “no difficulties” to “several difficulties”.

Decrease in financial conditions due to pandemic was assessed with a single item, where students were asked whether they perceived changes in the personal and family income in the last two years (referring to 2020–2022), in a 5-point rating scale, ranging from worsened considerably to improved considerably. This item was reverse scored.

### Ethics

Authorization to conduct the investigation was requested initially to the Ethics Committee of the Lusófona University, which was approved (Ref. CEDIC-2022-03-06). Before carrying out the protocol, all participants were asked to give informed consent, in which confidentiality, the use of data for research purposes only, and the option to withdraw at any moment during the study were presented. It also presented that privacy and data protection are in accordance with the General Data Protection Regulation (GDPR) of European Union, and all stored data will be kept for the necessary time until the study conclusion. In the online format, the participants read the consent and gave written consent. In the telephone format, the participants received the consent and gave it verbally. The interviewers were trained to read it to the participants, ask in the end if they understood it, if they wanted to hear it again and/or had any questions, and then ask for the consent. This survey was conducted in full agreement with the American Psychological Association Ethical Principles of Psychologists and the Code of Conduct [103].

### Procedure

Data collection took place between November, 2022 and February, 2023 via online and telephone. These two modalities were chosen to limit mode-specific effects, to ensure higher representativity of different segments of the population, and to broaden the participation of students with low technology accessibility. The protocol was exactly the same in both modalities. The telephone data collection was carried out using the CATI system (Computer Assisted Telephone Interviewing), by 27 interviewers with experience in telephone surveys. In each region, the interviews were distributed among several interviewers, to avoid a significant portion of the interviews being done by only one or two interviewers. As for the online data collection, this was done through an online survey directly accessed by the participants, who had previously been informed of this project through an email with information about it and the link to access the survey, through the CAWI system (Computer Assisted Web Interviewing). The abandonment rate was 6.8%. After the data collection (both online and by telephone) the questionnaires were reviewed, and possible errors or missing information were detected. On a case-by-case basis, an evaluation was made of the procedures to be adopted, which could range from a new contact with the respondent (to obtain the missing information) to the elimination of the interview (for example, if an abnormal non-response rate is verified in relation to the total number of questions). Of the total sample, 1002 resulted from online survey and 400 from telephone interviews.

## Data Analysis

Preliminary descriptive statistics and correlations were computed for all study variables using the Statistical Package for the Social Sciences [104]. Due to demographic variability in the sample, we tested whether dropout intention differed by gender, study cycle, residence status, and employment status. These comparisons, using independent samples t-tests, aimed to determine whether demographic controls were needed in the Structural Equation Modelling. Male students reported significantly higher dropout intention (*M *= 2.32, *SD *= 1.10) than females (*M* = 2.10, *SD *= 1.01), *t*(1307.52) = 3.89, *p* < .001, *d* = 0.21. Undergraduates also scored higher (*M* = 2.29, *SD *= 1.07) than master’s students (*M* = 2.04, *SD *= 1.01), *t*(1048.87) = 4.38, *p* < .001, *d* = 0.24. Displaced s*t*udents showed greater intention (*M* = 2.32, *SD *= 1.07) than non-displaced peers (*M* = 2.08, *SD* = 1.03), *t*(1400) = 4.33, *p* < .001, *d* = 0.23. No significant differences were found by employmen*t* status, *t*(1400) = −1.56, *p* = .118, *d* = −0.09, or institu*t*ion type, *t*(1400) = 0.98, *p* = .329, *d* = 0.07. Pearson’s correlation showed no significant associa*t*ion between age and dropout intention, *r*(1400) =.028, *p* = .290. Overall, gender, study cycle, and residence status were linked to meaningful differences in dropout intention and may warrant inclusion as controls in the modelling, while employment status, age, and institution type showed no substantial influence.

To assess the potential presence of common method bias, we conducted Harman’s single-factor test using maximum likelihood factor analysis. Results showed that a single factor accounted for only 24% of the total variance, which is well below the commonly accepted threshold of 50% [105]. Furthermore, model fit indices indicated poor fit for the single-factor solution (e.g., RMSEA = 0.169, TLI = 0.337, RMSR = 0.22), suggesting that common method variance is unlikely to be a serious concern in this study. To further examine the potential for common method bias, we tested an alternative model including an unmeasured latent method factor (ULMF), following the procedure recommended by Podsakoff et al. [105]. This method factor was specified to load equally on all indicators used in the measurement model, with one loading freely estimated for model identification. The model including the method factor showed a statistically significant improvement in fit compared to the baseline model without it (Δχ² [7] = 32.16, p < .001). However, the variance explained by the method factor was negligible, and all loadings were very small, with the factor’s variance being non-significant (p = .866). These results suggest that, although statistically detectable, likely due to the large sample size, the method factor does not meaningfully account for variance in the model. Therefore, these results indicate that common method bias is unlikely to pose a substantial threat to the validity of the observed relationships in this study.

Structural equation modeling (SEM) was conducted using R (v. 0.6–1) [106], specifically with the Lavaan Package [107]. The model was estimated using the robust maximum likelihood (MLR) estimator, which provides more accurate parameter estimates when data is ordinal and non-normally distributed [108]. Prior to model estimation, the distribution of the observed variables was examined. Skewness and kurtosis values were within acceptable thresholds (skewness ranging from −0.57 to 0.58; kurtosis ranging from −0.74 to −0.06), indicating no substantial univariate departures from normality. Furthermore, as several study measures were based on Likert-type items, the use of the MLR estimator was appropriate to account for both the ordinal nature of the data and any residual non-normality. This approach provides robust standard errors and adjusted fit indices, supporting the reliability of the model estimates. Fit indices reported (e.g., CFI, RMSEA, TLI) are the robust versions as returned by the Lavaan Package when using the MLR estimator.

To assess the goodness of fit of the hypothesised predictive model, we used the relative chi-square test, calculated by dividing the chi-square value by the degrees of freedom (χ²/df) [109]. While there is no universal consensus on an acceptable ratio for this statistic, recommendations vary, with suggested thresholds ranging from less than 5 [110] to less than 2 [111]. In addition, model fit was deemed acceptable if the Comparative Fit Index (CFI) [112] and the Tucker-Lewis Index (TLI) [113] were above.90, and if the Root Mean Square Error of Approximation (RMSEA) and the Standardized Root Mean Square Residual (SRMR) were both below.08 [114]. For model comparison, the Bayesian Information Criterion (BIC) and Akaike Information Criterion (AIC) were used, with the smallest value indicating the best-fitting model [115].

For a deeper understanding of the relationships between variables, we examined both direct and indirect effects in the SEM model. Direct effects represent the influence of an independent variable on a dependent variable without mediation, while indirect effects capture the mediated influence of an independent variable on a dependent variable through one mediator. These indirect effects were tested using bootstrapping procedures, generating confidence intervals to assess the significance of the mediation pathways [116]. Percentile bootstrap confidence intervals were used, as implemented in the lavaan package. These intervals are asymmetric by default. A statistically significant indirect effect suggests that the independent variable impacts the dependent variable through the mediator, offering deeper insight into the underlying causal pathways.

Effect sizes were interpreted using the standardized path coefficients (β), based on Cohen’s benchmarks for effect sizes in the behavioral sciences. In this framework, path coefficients were interpreted as small (β ≥ 0.10), medium (β ≥ 0.30), and large (β ≥ 0.50) effects [117]. Although originally developed for correlation coefficients, these thresholds are applicable in SEM contexts due to the conceptual and functional similarity between standardized path coefficients and partial correlations [118]. This approach has been adopted in recent SEM studies [119] to provide a heuristic interpretation of effect magnitude.

In addition, multi-group analyses were conducted to assess the robustness of the model across different groups (i.e., working vs. non-working students, and students living away from home vs. students living at home). Measurement invariance was tested by comparing models with increasingly restrictive constraints: configural invariance (which examines whether the factor structure is consistent across groups), weak invariance (which assumes equality of factor loadings across groups), strong invariance (which assumes equality of factor loadings and item intercepts), and strict invariance (which assumes equality of factor loadings, item intercepts, and measurement error variances across groups). While measurement invariance is often assessed in the literature using the change in chi-square (Δχ²) test, this approach is known to be highly sensitive to sample size, which can lead to over-rejection of the null hypothesis of invariance. To avoid these limitations, we opted for a more robust approach by examining changes in alternative fit indices, specifically the comparative fit index (CFI) and the root mean square error of approximation (RMSEA). A change (Δ) of less than 0.01 in these indices was taken as evidence of measurement invariance, while larger changes indicated potential non-invariance. Following the establishment of measurement invariance, we further tested for structural invariance to determine whether the structural paths (i.e., regression coefficients) were equivalent across groups. This was done by comparing a model in which the structural parameters were freely estimated across groups with a more constrained model in which these parameters were fixed to be equal. The comparison was conducted using the scaled chi-square difference test (Satorra-Bentler correction).

## Results

### Descriptive statistics and correlations

Table 1 presents the means, standard deviations, observed ranges, and bivariate correlations among the key study variables. Dropout intention (the outcome variable) was significantly correlated with all predictors and the mediator. Academic exhaustion (the proposed mediator) was significantly associated with all variables except course value.

**Table 1:** Means, standard deviations, minimum and maximum values, and correlations among variables.

Variable	M	DP	Min	Max	1	2	3	4	5	6	7	8	9	10	11	12	13	14
1 Pre-entry grade	15.42	2.27	9.5	20	–													
2 Satisfaction with education	3.59	0.93	1	5	0.03	–												
3 Course value	3.66	0.98	1	5	0.03	0.63^***^	–											
4 Academic Self-Efficacy	3.61	0.94	1	5	0.05	0.64^***^	0.75^***^	–										
5 Vocational difficulties	2.15	1.16	1	5	−0.02	−0.19^***^	−0.13^***^	−0.16^***^	–	–								
6 Social connectedness	4.29	1.25	1	6	0.01	0.13^***^	0.01	0.04	−0.34^***^	–	–							
7 Grade performance	2.03	.74	1	5	−0.12**	−0.130^**^	−0.094**	−0.22**	0.158^**^	−0.061^*^	–							
8 Social support	2.63	0.65	1	4.6	−0.01	−0.46^***^	−0.38^***^	−0.48^***^	0.28^***^	−0.39^***^	0.144^**^	–						
9. Institutional difficulties	2.19	1.08	1	5	0.01	−0.06^*^	0.05^*^	0.02	0.45^***^	0.17^***^	0.136^**^	0.24^***^	–					
10 Well-being	3.21	1.07	1	5	0.03	0.42^***^	0.35^***^	0.47^***^	−0.16^***^	−0.26^***^	−0.177^**^	−0.52^***^	−0.07^**^	–				
11 Autonomy difficulties	2.17	1.14	1	5	−0.05	−0.02	0.03	−0.08^**^	0.47^***^	−0.22^***^	0.114^**^	0.30^***^	0.39^***^	−0.15^***^	–			
12 Economic difficulties	2.47	1.22	1	5	−0.04	0.02	0.13^***^	0.06^*^	0.35^***^	0.13^***^	0.055^*^	0.23^***^	0.35^***^	−0.08^**^	0.46^***^	–		
13 Decrease in income	2.70	1.01	1	5	−0.02	−0.17^***^	− 0.09^**^	− 0.11^***^	0.11^***^	0.42^***^	−0.026	0.23^***^	0.11^***^	− 0.21^***^	0.13^***^	0.19^***^	–	
14 Academic exhaustion	2.78	0.98	1	5	0.04	−0.06^*^	−0.01	−0.10^***^	0.40^***^	−0.40^***^	0.129^**^	0.38^***^	0.37^***^	−0.24^***^	0.35^***^	0.30^***^	0.17^***^	–
15 Dropout intention	2.20	1.06	1	5	−0.02	−0.20^***^	−0.23^***^	−0.24^***^	0.49^***^		0.134^**^	0.41^***^	0.30^***^	−0.24^***^	0.35^***^	0.24^***^	0.15^***^	0.57^***^

*Note*. ^*^p < .05, ^**^p < .01, ^***^p < .001.

### Measurement model evaluation

To evaluate the adequacy of the measurement model, a confirmatory factor analysis (CFA) was conducted including all latent constructs used in the structural model. The model showed poor fit to the data: χ²(874) = 5502.88, CFI = .840, TLI = .827, RMSEA = .068, and SRMR = .106. Examination of the parameter estimates revealed that the construct Social Support presented multiple issues, including low standardized loadings, several nonsignificant indicators, and high residuals. To improve model parsimony and fit, this construct was removed. The revised model demonstrated a substantially improved fit: χ²(356) = 1111.12, CFI = .960, TLI = .955, RMSEA = .043, and SRMR = .033. Moreover, information criteria favored the revised model (original: AIC = 168797.46, BIC = 169405.96; revised: AIC = 110321.95, BIC = 110736.35), supporting the decision to exclude the problematic construct. These results indicate that the revised measurement model provides a more parsimonious and statistically adequate representation of the latent constructs used in the final model.

### Structural equation modeling

Structural equation modeling (SEM) was employed to investigate the factors associated with ‘s intention to dropout. The observed variables included family educational level, pre-entry grade, decrease in financial conditions due to pandemic, economic difficulties, whether the course was the first option, course value, vocational difficulties, grade performance, difficulties in adaptation to the academic institution, and autonomy difficulties. The latent variables were social connectedness to the campus, well-being, satisfaction with education, and academic self-efficacy. Academic exhaustion was used as a mediator. Gender, study cycle (undergraduate vs. master’s), and residence status were included as control variables.

The structural equation model was estimated excluding the Social Support latent construct, which had shown poor psychometric properties in the measurement model. The model demonstrated good model fit: χ²(680) = 2124.61, p < .001; CFI = .934; TLI = .926; SRMR = .075; robust RMSEA = .042 [90% CI:.040,.044]; AIC = 109,962.78; BIC = 110,503.08. However, two predictors: perceived grade performance and whether the course was the student’s first option did not significantly contribute to the prediction of either academic exhaustion or dropout intention. Residence status was also excluded as a control variable, as it did not show significant associations with either the mediator or the outcome in the original model. A revised nested model was therefore specified, removing the two non-significant predictors and residence status, while retaining the control variables gender and study cycle.

The nested model demonstrated slightly improved fit: χ²(599) = 1940.29, p < .001; CFI = .937; TLI = .930; robust RMSEA = .043; SRMR = .078. Information criteria were also lower (AIC = 109,959.73; BIC = 110,468.56). A Satorra-Bentler scaled chi-square difference test confirmed that the nested model provided a significantly better fit to the data than the full model, Δχ²(81) = 175.47, p < .001. These results support the more parsimonious model.

**Fig. 1:**
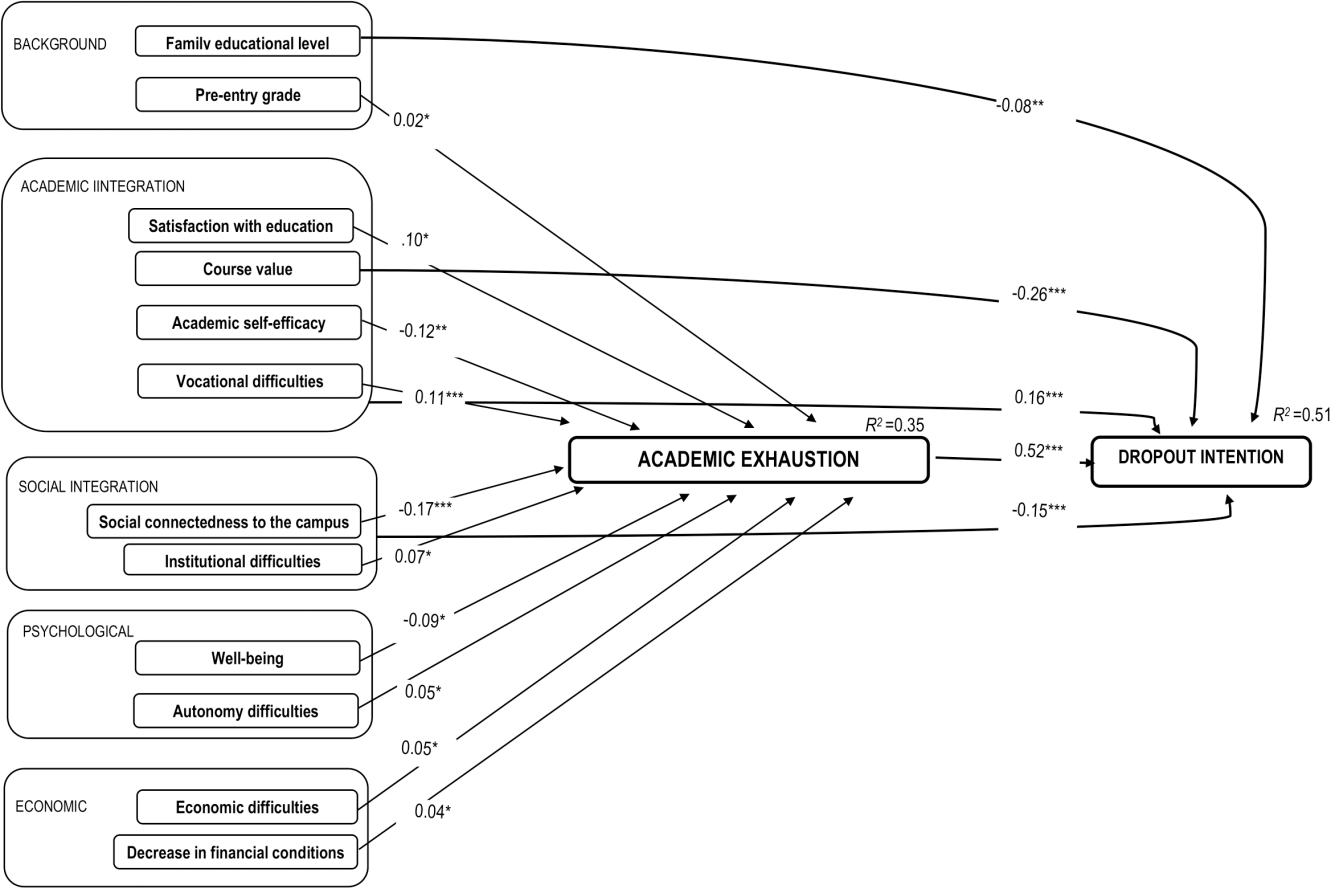
Path model testing the direct and indirect effects on dropout intention, via academic exhaustion. Only significant paths are represented. *Note*.***p < .001; **p < .01; *p < .05.

The direct and indirect effects of the revised model are presented in Table 2 and illustrated in Fig 1. The strongest predictor of dropout intention was academic exhaustion, with a large direct effect (β = 0.523). Social connectedness to campus showed both direct (β = −0.145) and indirect (β = −0.165) effects, resulting in a total medium effect (β = −0.310). Vocational difficulties also showed both direct (β = 0.162) and indirect (β = 0.112) effects, summing to a total effect of β = 0.274, which is interpreted as small. Course value had a small direct effect (β = −0.256), though its indirect effect was not significant. A small direct effect was also found for family educational level (β = −0.080). Small but significant indirect effects, through academic exhaustion, were observed for satisfaction with education (β = 0.097), academic self-efficacy (β = −0.117), well-being (β = −0.091), institutional adaptation difficulties (β = 0.069), economic adaptation difficulties (β = 0.049), autonomy difficulties (β = 0.049), pre-entry grade (β = 0.016), and decrease in financial conditions (β = −0.035).

**Table 2:** Confidence intervals of standardized total, direct, and indirect effects for the final model.

Model routes	Total effects 95% CI	Direct effects 95% CI	Indirect effects 95% CI
Academic Exhaustion → Dropout Intention	—	0.523 [0.488, 0.684]	—
Social Connectedness to the Campus → Academic Exhaustion → Dropout Intention	−0.310 [−0.300, −0.181]	−0.145 [−0.163, −0.059]	−0.165 [−0.170, −0.093]
Satisfaction with Education → Academic Exhaustion → Dropout Intention	0.155 [0.045, 0.280]	0.058 [−0.042, 0.167]	0.097 [0.032, 0.174]
Academic Self-efficacy → Academic Exhaustion → Dropout Intention	−0.089 [−0.262, 0.071]	0.028 [−0.123, 0.187]	−0.117 [−0.215, −0.040]
Course Value → Academic Exhaustion → Dropout Intention	−0.196 [−0.389, −0.036]	−0.256 [−0.450, −0.116]	0.060 [−0.022, 0.154]
Well-being → Academic Exhaustion → Dropout Intention	−0.111 [−0.172, −0.035]	−0.020 [−0.080, 0.046]	−0.091 [−0.129, −0.050]
Institutional Difficulties → Academic Exhaustion → Dropout Intention	0.037 [−0.023, 0.087]	−0.032 [−0.083, 0.025]	0.069 [0.030, 0.092]
Economic Difficulties → Academic Exhaustion → Dropout Intention	0.091 [0.027, 0.117]	0.042 [−0.017, 0.079]	0.049 [0.012, 0.064]
Autonomy Difficulties → Academic Exhaustion → Dropout Intention	0.105 [0.030, 0.136]	0.056 [−0.004, 0.096]	0.049 [0.012, 0.069]
Vocational Difficulties → Academic Exhaustion → Dropout Intention	0.274 [0.164, 0.279]	0.162 [0.072, 0.187]	0.112 [0.062, 0.124]
Family Educational Level → Academic Exhaustion → Dropout Intention	−0.062 [−0.134, −0.015]	−0.080 [−0.151, −0.044]	0.018 [−0.011, 0.053]
Pre-entry Grade → Academic Exhaustion → Dropout Intention	0.011 [−0.017, 0.027]	−0.024 [−0.028, 0.009]	0.016 [0.003, 0.035]
Decrease in Financial Conditions → Academic Exhaustion → Dropout Intention	0.043 [−0.011, 0.089]	0.008 [−0.035, 0.051,]	0.035 [0.003, 0.062]

*Note*. Gender and study cycle were included as control variables. Values represent standardized estimates. Confidence intervals are presented in brackets.

The model explained 51% of the variance in dropout intention and 35% of the variance in academic exhaustion. The total effects were statistically significant, β = 0.398, p < 0.001. The confidence intervals of standardized total, total indirect, specific indirect, and direct effects are shown in Table 2.

### Multi-group analyses

We conducted a multi-group analysis within the SEM framework to examine sociodemographic differences in the nested model. To evaluate whether the measurement model was invariant across different groups (working vs. non-working students, and students living away from home vs. students living at home), we tested for configural, weak, strong, and strict invariance. The results showed acceptable model fit across all levels, with changes in CFI and RMSEA between increasingly constrained models remaining below the recommended thresholds (ΔCFI ≤ .01; ΔRMSEA ≤ .01). Although some chi-square difference tests reached significance, the invariance criteria were met based on changes in fit indices, which are more robust to sample size. This supports the assumption of measurement invariance, indicating that the latent constructs are measured equivalently across groups (Table 3), thus allowing meaningful comparisons.

**Table 3:** Significance Values of Multi-Group Analyses.

Group		Df	CFI	RMSEA	AIC	BIC	χ² diff (SB)	Df diff	Pr(>Chisq)
Working	Configural	712	0.934	0.046	110426	111559	—	—	—
	Weak	734	0.933	0.045	110420	111437	40.42	22	0.0097^**^
	Strong	756	0.933	0.045	110409	111311	33.26	22	0.0583
	Strict	785	0.932	0.045	110399	111149	28.28	29	0.5031
Displaced	Configural	712	0.938	0.044	110363	111496	—	—	—
	Weak	734	0.937	0.044	110356	111374	39.06	22	.0139^*^
	Strong	756	0.937	0.044	110330	111233	18.17	22	.6962
	Strict	785	0.937	0.043	110369	111119	57.93	29	.0011^**^

*Note*. CFI = Comparative Fit Index; RMSEA = Root Mean Square Error of Approximation; AIC = Akaike Information Criterion; BIC = Bayesian Information Criterion.

χ² diff (SB) = Scaled chi-square difference based on the Satorra-Bentler method.

Significance codes: ***p < .001; **p < .01;· *p < .05. A ΔCFI ≤ 0.01 and Δ RMSEA ≤ 0.01 indicates that the null hypothesis of invariance cannot be rejected.

Following the confirmation of measurement invariance, we tested for structural invariance to examine whether the relationships between predictors, the mediator, and the outcome were equivalent across groups (e.g., working vs. non-working students). Specifically, we compared a model where structural paths were freely estimated across groups to a model in which all regression paths were constrained to be equal. The scaled chi-square difference test (Satorra-Bentler) revealed no significant difference between the unconstrained and constrained models, Δχ²(29) = 32.30, p = .307. These results support the equivalence of the structural model across groups, indicating that the predictive relationships are consistent regardless of students’ work status. The same result was observed for residence status, with the constrained model not differing significantly from the unconstrained model, Δχ²(29) = 40.82, p = .071, indicating structural invariance across this grouping variable.

## Discussion

Focusing in a particularly vulnerable period of dropout – the post-pandemic period-, this study’s main goal was to test a predictive model of dropout intention, examining the links between five different domains of variables (background, academic integration, social integration, psychological and economic variables) and dropout intention from HE, and whether these links were mediated by academic exhaustion, in a sample of Portuguese HE students. Our first hypothesis was substantially supported, as 12 of the 15 integrated variables were found as significant predictors of dropout intention. Our second mediational hypothesis received partial support. Results showed that most of the variables had an indirect effect on dropout intention, through academic exhaustion. Family educational background and course value only had a direct effect on dropout intention. Satisfaction with social support, grade performance, and course as first option did not have any significant effects on dropout intention. Finally, our last hypothesis was supported, as academic exhaustion was the strongest predictor of dropout intention, the only predictor with a large effect size. Exhaustion is the core of academic burnout [88], which occurs when students feel overwhelmed without having (or feeling they do not have) the effective resources to face prolonged stressful events [84]. According to Turhan [8], theoretical models for dropout should explicitly include burnout symptoms, which are considered the strongest determinants of actual dropout decisions. Marôco et al. [65] has found that although both engagement and burnout are good predictors of subjective academic performance and dropout intention, burnout suppresses the effect of engagement on these variables. Therefore, preventing dropout requires more than just promoting engagement—importantly, burnout levels must also be kept low. As academic exhaustion is considered an early indicator of burnout [86,120], its quick detection could help provide timely support to students. This is crucial, as academic exhaustion not only negatively affects students’ health and academic performance during their studies [86] but also increases their susceptibility to burnout in the workforce [121].

Social connectedness to the campus was the second strongest predictor of dropout intention, with a medium effect, both directly and through academic exhaustion. Our results are in line with the ones of Piepenburg et al. [43], which showed that social integration with fellow students and vocational issues are amongst the strongest predictors of dropout intention. Regarding social connectedness to campus, the need to form and maintain positive interpersonal relationships – the need to belong – is a basic and pervasive human motivation, and people seek environments that fulfil this need [122]. University connectedness has been associated with less dropout [66,123,124], increased class attendance, more academic engagement [63], improved academic achievement, a stronger sense of efficacy and competence in studying, greater motivation to study, and more time devoted to studying [125]. Moreover, when students feel less connected to the institution, peers, and teachers, they show more signs of academic exhaustion [126]. On the other hand, when the academic load is high and the student is facing academic challenges, having good social connections with peers may help feel less exhausted and with more strength to pursue studying [125]. It is plausible that the pandemic context, with interruption of in-person classes and contact limitations has hindered this sense of connection, thus affecting academic exhaustion and the desire to persist in HE. First-year students may have missed the usual academic and social reception, thereby losing an initial opportunity to connect with their peers. Promoting a sense of connectedness might be a protective factor that universities could use to protect students’ mental health, especially nowadays, with the increase of mental health concerns in HE students since the pandemic [72,73,127].

The next strongest predictors of dropout intention were vocational difficulties, although with a small effect only, both direct and indirect, and course value, with a small direct effect. Some studies have shown the relation of vocational difficulties with dropout intention [43,45,52,62,128,129]. George et al. [34] has found that students contemplate dropping out primarily because of weak commitment to their course of study in general, or to the specific field of study. Rahmatpour et al. [130] have also found a positive association of vocational difficulties with academic burnout, although not specifically with academic exhaustion. Unfulfilled or unrealistic expectations concerning the course/field of study are dropout intention predictors [5,49,131]. Our results align with career theories that emphasize the importance of matching students’ interests with their chosen fields of study or future career paths in promoting study satisfaction and retention [132]. Pertaining to course value, former studies have found this association with dropout intention [5,44,60,61]. According to students’ goals and values, the more they perceive their course as useful—i.e., aligned with their personal future goals and values—the more likely they are to persist, even in the face of difficulties and challenges. Therefore, the direct association with dropout intention is reasonable: if students do not perceive their course as valuable, they may be more likely to withdraw, even in the absence of academic exhaustion and, in the same vein, they are more likely to persist, even in case of exhaustion.

Autonomy difficulties, which had a small indirect effect on dropout intention, have been shown to be a dropout intention predictor [45,76]. Regarding the transition to higher education, a qualitative study [76] identified a key theme related to the challenges of independent living, including adapting to a new environment, managing new domestic responsibilities, and a lack of preparedness for everyday tasks. These challenges and feelings of unpreparedness can be particularly distressing for students, as they may perceive their early academic performance as directly linked to their future success at HE and beyond. Regarding the indirect path through academic exhaustion, a possible explanation is that students with low autonomy have fewer tools to cope with the multiple demands of the academic environment. Their inability to manage time effectively, even for basic daily tasks, likely results in reduced efficiency and increased exhaustion.

Two of our background variables had a small effect on students’ dropout intention: family educational level, directly; and pre-entry grade, indirectly. Our results are consistent with previous studies that point to the importance of students’ socio-educational background in HE attendance, persistence and dropping out [2,6,28,133]. In Portugal, most students come from non-tertiary family educational backgrounds. Given the well-established association between parental education level and family financial status [133], this issue is particularly sensitive, as families remain the primary source of financial support for students, followed by students’ own earnings and scholarships [28]. Beyond prolonging financial dependence on their parents, some students may fear overburdening their families [28], leading them to leave higher education. These findings highlight the risk of perpetuating social inequalities in education. The other background variable, pre-entry grade, had a small indirect effect on dropout intention. With the mediation of academic exhaustion, studies have found that students who enter HE with lower prior grades are at a greater risk of dropping out, by feeling more exhausted with the academic demands and tasks [64,65]. Sosu et al. [79] have found that students with the lowest entry grades were about 2.17 times more likely to dropout.

Considering the academic integration variables, the more satisfied with the education the students are, the higher the dropout intention, in a relation totally mediated by academic exhaustion. This result seems unexpected, considering the results of other studies, which showed that more satisfied students with features as classes organization, schedules, and teacher availability, were less exhausted and less prone to abandon HE [13,46]. However, previous studies [41] looked at the period before the pandemic and only included first-year students. Our study is set in the period after the pandemic, and covers several years of HE. These differences may be relevant, and future studies should investigate this relation. Marôco et al. [65] interpreted that engagement may lead to burnout, which in turn can affect academic performance and dropout, possibly when engagement is associated with personality features like perfectionism. Considering the literature on burnout in the work field, studies in different populations show that higher expectations and goals in respect to the employees´ goals lead to higher efforts and thus to higher emotional exhaustion [134]. Thus, a possible interpretation is that students who are more satisfied with their education may also be more academically engaged and invest greater effort in their studies, which could lead to increased exhaustion and, ultimately, a higher intention to dropout.

Pertaining to academic self-efficacy, the students’ trust in their abilities to tackle their course successfully is considered an important determinant of dropout intention [51,52,54]. In our study, the effect of academic self-efficacy was totally mediated by academic exhaustion. Previous studies show that burnout (integrating professional efficacy, emotional exhaustion, and cynicism) predicts dropout intention [8,65,80]. Therefore, it is reasonable that low academic self-efficacy contributes to academic exhaustion, which, in turn, increases the likelihood of dropout intention. This suggests that dropout intention emerges indirectly, as students feel increasingly overwhelmed by academic tasks and demands, rather than as a direct consequence of low academic self-efficacy.

Difficulties in adapting to HE institution has an indirect effect on dropout intention, which is congruent with the literature. Dissatisfaction in the contacts with teachers and members of the HE institution leads students to reduce their participation in campus activities, which negatively impacts interpersonal relationships and the construction of social support networks [41], as well as academic achievement [135]. Reduced positive cognitive and emotional experiences in academic contexts tend to heighten feelings of academic exhaustion over time [8], as well as academic disengagement [136], and are associated with the dropout intention [8,137].

The effect of the psychological variable of well-being in dropout intention is also indirect, through academic exhaustion. Lower levels of well-being are predictors of dropout [21,138]. Students who experienced remote teaching in the pandemic reported significantly higher levels of negative emotions (e.g., anxiety, boredom, stress) [139,140]. Schriek et al. [13] have found that low levels of well-being are associated with higher academic exhaustion, leading students to feel more vulnerable and prone to abandon HE.

Finally, the economic variables only showed a small indirect effect on dropout intention, but not a direct one. European students reported a (very) negative impact of the pandemic on the financing of their studies [28]. For students from low-income families, fees can cause or at least exacerbate financial difficulties, which can influence their commitment to stay in HE, which can lead to a higher risk of dropping out [141]. It is important to note that students from lower socioeconomic backgrounds more often pursue their studies with lower intensity and part-time jobs, experiencing difficulties completing their degrees on time, even after controlling for academic performance, educational behaviors, program characteristics, and institutional characteristics [142]. Several authors state that economic variables may have an indirect effect on academic issues [16,40,43], though less than social and academic integration. Economic difficulties make daily living more unstable, leading to less focus on studying, which brings greater exhaustion [64]. Thus, financial aid per se is not enough to influence HE persistence decisions, but the indirect nature of the influence of finances on other academic and social facets of the ‘s education must be kept in mind by policymakers and institutional administrators [16]. However, and considering that vocational difficulties and course value were among the highest determinants of dropout intention, although with a small effect, it is important to keep in mind that especially among low-income students, high course value may lead students to persist even if they must adopt strategies such as working while studying. By contrast, even in case of little financial pressure, students may choose to leave because HE is not that valued [16]. There is another feature that may help explain indirect effects only on dropout intention. Although 43.6% of students reported that their personal or family financial situation worsened due to the pandemic, it is plausible that those facing these difficulties carefully chose to enroll in HE only if they believed they could afford to sustain their studies. Therefore, economic difficulties did not lead to dropout intention directly, but only through a progressive feeling of exhaustion.

Two academic variables of our model, course as first option, and grade performance, did not present significant effect on dropout intention, either directly or indirectly, through academic exhaustion. Despite the evidence of the association between attending a non-first option course and higher dropout intention [41], it is possible that these choices do not necessarily reflect students’ initial preferences and may not be entirely well-founded (e.g., due to low autonomy, economic difficulties, or insufficient grades), which could limit the protective role of a first-choice course attendance against dropout intention. In countries like Portugal, the admission in HE institutions is determined by the seriation of candidates based on the access marks, and therefore the access of students to the course of their intrinsic preference is many times conditioned [48]. Also, the fact that grade performance has no effect on dropout intention is not in line with the literature, as several studies show this effect [55]. Nevertheless, this perception is shaped by personal comparisons, which are influenced by the reputation of the academic institution (higher or lower) and the course’s selectivity (whether it requires high entrance grades or not). Academic grades vary significantly across fields of study and institutions, giving them different meanings for each specific situation. For example, the same absolute grade may be perceived as either good or poor, depending on the field of study and the educational institution [43]. Also, the potential effect of this variable may largely depend on students’ personality traits, such as perfectionism and self-demand levels. Moreover, this study suggests that course value and vocational difficulties had a stronger impact on dropout intention than students’ self-perceived academic performance.

The results of the present study are strengthened by the identification of the model’s invariance across students working status and living situation. This model integrates relevant variables with most of them relating to dropout intention through academic exhaustion. Most literature points that both working students and students living away from home face more challenges in HE, with higher dropout rates in working students [5,28,92–95] and higher risk of dropping out in students living away from home [41,79]. However, some studies showed that having a job was not a major factor in a student’s dropout intention, being important to comprehend the significance of securing a job as part of the educational process [67]. Moreover, working students often reveal more developed soft skills, such as communication or time management skills, acquired from their experience in the labour market, which partly countervails the negative effect caused by less time to study [143]. Pertaining to students living away from home, Cocorada et al. [51] didn´t find a higher risk of dropping out in these students, and Toyon [67] found that students’ living situation has a smaller effect on dropout intention. Differently from suggested by some previous research [40,67,95], social integration variables, namely difficulties in adaptation to the academic institution, which in our study included relation with teachers and staff, were not stronger predictors of dropout intention for working-students. In fact, social connectedness to the campus was the second stronger predictor of dropout intention irrespectively of students´ working or living status.

Our results seem to indicate that interventions to prevent dropout intention should address the variables which showed significance in this model, irrespective of the working and living situation of HE students.

### Practical implications

In order to prevent or alleviate the phenomenon of HE dropout, some recommendations may stem from our results. Greater attention should be given to early indicators of academic exhaustion – considered the first stage of burnout syndrome or the first warning sign [8]. Extreme tiredness, absenteeism or late submission of study work because of the tiredness, and an overall reduced energy level can be considered first signs of academic exhaustion. To the best of our knowledge, addressing students’ academic exhaustion has played only a minor role in efforts to reduce dropout rates, despite the existence of initiatives such as workshops and webinars on mental health and time management. Major efforts (e.g., reducing course-work load or increased coordination between teachers of different subjects) must be pursued to prevent burnout. Following the Model Job Demands- Resources Model [144], where burnout symptoms emanate as a result of ongoing loss of resources, it is important to develop guidelines that would make available resources like teacher support, peer support, motivational, and behavioral self-regulation learning strategies for coping with challenging academic demands and stressors. Besides, teachers must ensure that demands are balanced and can be mastered in appropriate time spans. These recommendations may be more likely to be implemented, as they do not demand more financial resources. In order to counteract emotional exhaustion, clinical psychologists could apply interventions that previously showed effectiveness in reducing psychological distress and enhancing relaxation, namely Mindfulness-Based Stress Reduction interventions [145]. Given the persisting stigma surrounding mental health interventions, it is essential to frame these programs within the context of health promotion and prevention [68,72]. Moreover, the Implementation of these measures may face resistance, especially in education systems hesitant to invest further—such as in countries like Portugal, where education already consumes a large share of GDP per capita [29]. Given the impact of social connectedness to campus on dropout intention, strengthening and improving mentorship programs—if broad and well-designed—could play a crucial role in preventing academic dropout. These programs should not only support students academically but also foster social integration, helping them feel accepted and more connected to the campus community.

As our data suggests that dropout may take place throughout the whole trajectory in the HE, mentorship should cover all years and not only the first year. Regarding difficulties in vocational adaptation, Ortiz and Dehon [141] draw attention to the importance of developing vocational reorientation activities as soon as students begin to show signs of academic difficulty – ideally before the end of their first year. They showed that after a failed year, a significantly higher proportion of students who re-enrol in a different field obtain a degree compared to those that re-enrol in the same field, suggesting that universities should rethink the mechanisms available to manage failure and guide students’ choices. In the same line, to prevent students from having unmet expectations about potential growth opportunities in their chosen fields of study, academic institutions could take proactive measures, as creating recruitment materials that accurately portray the institution and its academic programs. Additionally, universities could develop online self-evaluation tools that allow prospective students to assess their compatibility with specific programs [146]. Such initiatives could help align students’ expectations with the actual offerings of their selected courses of study. Career guidance programs play a critical role in helping students understand their personal characteristics and make informed career choicest that align with their values, interests, and abilities [147]. To decrease vocational adaptation difficulties, a program could be developed within the academic institutions, in which students may have practical experiences and observation traineeships, enhancing the link between theory and practice in their vocational field. Moreover, transition to HE should be prepared with greater articulation between HE and high school institutions, with vocational orientation being reinforced in the last years of high school. The implementation of utility value interventions, pertaining to the course the students are attaining, could also be considered, as it has been shown that brief utility value interventions have relevant effects, such as subsequent course choice, and career aspirations in college, not only on short term (within a semester) but also for longer [148].

Finally, frequent evaluation of current interventions and support services, together with satisfaction surveys, can inform improvements and modifications aimed at better meeting students’ changing needs. We acknowledge that institutional barriers, concerning resistance to innovation and making changes, may be very strong, even if no significant financial resources are involved. Overall, within the scope of the higher education system organization of each country, the degree of institutional autonomy is a crucial variable that may facilitate or, on the contrary, be a huge barrier to the proposed changes, i.e., in countries as the United States and UK, with a decentralized HE system [27], granting significant autonomy to institutions, the possibility of each institution to develop and adopt the proposed measures is certainly much higher than others.

### Limitations and future studies

The findings of this study should be interpreted with certain limitations in mind. While we used dropout intention as a proxy for actual HE dropout, this does not provide a direct measure of dropout rates. Only a longitudinal study could determine how many participants would ultimately dropout HE. Time and budget constraints precluded the operationalization of the stratified random sampling method initially planned. Despite the multisite sampling process adopted, the convenience quota method used in data collection as a non-random method prevented the representativeness of all students, potentially limiting the external validity of the results. The cross-sectional research design precluded the establishment of causality among study variables. The issue of variable changes over time was not addressed. While our sample comprises students from different academic years, future longitudinal research may account for the possible trajectories of certain variables (e.g., social connectedness) to dropout decisions since changes are expected throughout the HE experience. In the present study we focused on dropout intention as the intention to leave HE before earning a degree. Therefore, results may not account for situations where students may choose to transfer to another institution, decide to change major or leave HE only temporarily. Although we relied solely on self-reported data, the used procedures to assess common method bias showed no indication of that bias significantly influencing the results. Finally, the satisfaction with social support could not be included in our measurement model due to poor psychometric performance, but it should be included in future studies, as the literature suggests possible relevance of extra academic social support in the dropout process, namely from families [79].

Several challenges in the field of HE are shared by different countries, which makes the prevention of dropout such a relevant topic. Namely, young people generally receive diminished rewards compared to past generations [57], and although many young adults in Western countries still recognize the value of obtaining a degree [149], an increasing number are questioning the value of higher education, particularly as a pathway to high-paying jobs and a fulfilling life [65,150]. In some European countries, including Portugal, younger adults with a bachelor’s or equivalent degree have higher inactivity rates than the ones who completed a vocational upper secondary or post secondary non-tertiary program [29]. However, while we consider the findings of this study may be transferable to other HE contexts—particularly within the European Union—it is important to recognize the limitations of such generalizations, concerning the relevance of national level determinants of dropout [2]. Although general research on HE dropout points in the direction that there are similarities at least across Europe and the USA, variable factors such as educational systems financing policies and models, fees, and access modalities should be considered when interpreting the results for another context. Countries differ significantly in their higher education funding models, student support structures, and social safety nets [2,27]. For instance, in countries like USA or UK where tuition fees are higher or where student financial aid may be less accessible, economic stressors may exert a more direct influence on dropout intentions, potentially bypassing academic exhaustion as a mediating mechanism. Conversely, in contexts with robust financial and psychological support services, the impact of factors like vocational misfit or academic workload might be mitigated. These contextual differences may alter the strength or pathways of the relationships observed in our model. Therefore, while the structural robustness of the model across subgroups in our sample suggests some generalizability, caution is warranted in applying these findings universally. Cross-national comparative studies are needed to test the model’s applicability in varying socio-economic and institutional frameworks.

Furthermore, the fact that the effect of satisfaction with education on dropout intention was fully mediated by academic exhaustion, may raise the focus on the increased susceptibility of certain people to suffer from academic exhaustion. In face of the relevance of personality characteristics, as perfectionism [151] or conscientiousness [152], future studies should analyse personality variables and its relationships with academic exhaustion and dropout intention.

Finaly, it is known that public health measures to contain Covid-19 pandemic effects impacted the students` satisfaction with the education, performance and psychological well-being [7,9–11]. Long before the pandemic, post-graduation outcomes tend to remain highly stratified by social class and other markers of privilege [57], and the pandemic may have heightened pre-existing financial and education disparities, as well as inequality of opportunities for success in HE [14,28].Therefore, further research is needed to understand if the impact on mental health and well-being, on the one side, and on inequality of opportunities, on the other, decreased over time and/or with government support measures, or not, and its relation with dropout rates in the following years.

## Conclusions

Despite the above limitations, this study is innovative not only because it focuses on a particularly vulnerable timing of dropout (post-pandemic era), but also by acknowledging the multi-domain complexity of the dropout phenomena, unravelling new pathways of influence, through academic exhaustion. Using a large and diverse sample that reflects the main socio-demographic and academic characteristics of the Portuguese higher education population, the results highlight the significance of individual factors—such as academic exhaustion and lack of fit with the course—in the dropout decision-making process. At the same time, they underscore the crucial role of academic institutions and the broader education system in addressing this issue, particularly in areas such as academic workload, vocational guidance, social environment, mental health service accessibility, and financial support. Model invariance across two sub-groups, based on working and residence status, further reinforces the relevance of the selected dropout intention predictors. It also highlights the crucial mediating role of students’ academic exhaustion in the dropout process. Even though the results in the present study were obtained using data from Portuguese HE institutions, we believe that they are extendable to other countries with similar HE contexts, especially in the EU, where the suggested policy recommendations may also be relevant and effective.

## Supporting information

S1 Data Dataset. (SAV)
